# Plasma lipid levels are associated with the CD8+ T‐cell infiltration and prognosis of patients with pancreatic cancer

**DOI:** 10.1002/cam4.6080

**Published:** 2023-05-15

**Authors:** Bin Wu, Weiai Shen, Xiaoguang Wang, Jing Wang, Zhengxiang Zhong, Zhongchen Zhou, Xujian Chen

**Affiliations:** ^1^ Department of Hepatobiliary Surgery The Second Affiliated Hospital of Jiaxing University Jiaxing China; ^2^ Medical College of Ningbo University Ningbo China

**Keywords:** cholesterol, immunotherapy, lipid, pancreatic cancer, triglycerides, tumour‐infiltrating lymphocyte

## Abstract

**Background:**

Recently, researchers have found that the tumour microenvironment plays an important role in tumours. We aimed to investigate the effects of plasma lipids on the prognosis of patients with pancreatic cancer and the infiltration of CD8+ T lymphocytes in tumour tissue.

**Methods:**

We enrolled patients with pancreatic ductal adenocarcinoma (PDAC) who underwent pancreatic surgery between 2012 and 2021. Clinical pathological data were recorded; total cholesterol (TC) and triglyceride (TG) levels were measured; and tissue samples were collected. A tissue microarray was generated using collected tissue samples, and CD8 staining was performed to determine immunoreactive scores (IRSs). The correlations of TC and TG levels with clinicopathological characteristics and prognosis were analysed. Finally, the correlations of TC and TG levels with CD8+ T‐cell infiltration were analysed.

**Results:**

A total of 90 eligible PDAC patients were included. TC levels were significantly correlated with tumour grade and lymph node metastasis, and TG levels were significantly correlated with perineural invasion. The Kaplan–Meier survival analysis results indicated that the prognosis in the high TC group was significantly worse than that in the low TC group, and the prognosis in the high TG group was significantly worse than that in the low TG group. Cox multivariate analysis indicated that TC was an independent indicator of poor prognosis of pancreatic cancer patients after surgery. Spearman correlation analysis indicated that there were significant negative correlations between CD8 IRS and TC and between CD8 IRS and TG.

**Conclusions:**

TC and TG levels are significantly related to the prognosis of pancreatic cancer patients. They may be associated with tumour progression to higher grades, lymph node metastasis and/or nerve invasion. More importantly, TC and TG may reduce CD8+ T lymphocyte infiltration into pancreatic cancer tissue, affecting antitumour immune functions and immunotherapy efficacy.

## 1 INTRODUCTION

Pancreatic cancer is a highly malignant tumour and is currently the third leading cause of cancer‐related death.^1^ Recently, the incidence rate has increased each year. Surveillance, Epidemiology, and End Results data suggest that pancreatic cancer will continue to be the leading cause of cancer‐related death.^2^ However, although there have been continuous breakthroughs and innovations in the field of cancer treatment, pancreatic cancer treatment is not as effective as treatment for other tumours and patient prognosis has not significantly improved. The 5‐year survival rate for patients with pancreatic cancer is only approximately 10%.^3^ The surgical resection rate of pancreatic cancer is very low, and the efficacy of traditional radiotherapy and chemotherapy is poor. Recently, immunotherapy has achieved good outcomes for some tumours, including melanoma, breast cancer and lung cancer.^4^ However, the efficacy of immunotherapy in pancreatic cancer appears to be poor. What causes the poor response of pancreatic cancer to immunotherapy? Recently, researchers found that the tumour microenvironment plays an important role in tumours, potentially affecting the efficacy of immunotherapy. The tumour microenvironment can regulate the malignant biological behaviour of tumours, and immunotherapy targeting the tumour microenvironment is feasible. Therefore, it is necessary to explore the immune microenvironment of pancreatic cancer and its influencing factors.

In addition to tumour cells, the tumour microenvironment also includes interstitial cells, various immune cells and various metabolic substances. Tumour‐infiltrating immune cells, including T lymphocytes, B lymphocytes, macrophages, neutrophils and dendritic cells, are important for antitumour immune responses. Among them, CD8+ T lymphocytes play a major role in killing tumour cells. Researchers have proposed different categories of tumour immune phenotypes/immunophenotypes based on the infiltration of immune cells into tumour tissue.^5^, ^6^ Tumour immune cell infiltration is divided into several categories. (1) In cases of inflammatory infiltration, there is a large amount of CD8+ T‐cell infiltration in the tumour parenchyma, with immune cells in close proximity to the tumour cells.^7^ (2) In cases of immune exclusion, there is relatively abundant immune cell infiltration around the tumour, with immune cells unable to enter the cancer nest but in the surrounding stroma^8^, ^9^; in patients with this phenotype, the infiltration of T lymphocytes into cancer nests may be limited due to immunosuppressive factors in the tumour microenvironment. (3) In immune desert cases, there is a lack of T‐cell infiltration in the tumour parenchyma and stroma,^8^, ^10^ and such tumours are also considered noninflammatory tumours. Previous studies and our preliminary studies have shown that the tumour‐infiltrating lymphocytes (TILs) that play the largest role in antitumour immunity are the CD8+ T cells that infiltrate cancer nests; however, there may be insufficient numbers of CD8+ T cells in the stroma of tumour nests.^11^ In such cases, reduced CD8+ T‐cell infiltration and activity may be the main reasons for nonresponse to immunotherapy.

Previous studies have shown that lipids are closely related to tumours and are likely to affect tumour immunity. Studies have found that the inhibition of cholesterol esterification enhances the antitumour activity of CD8+ T cells.^12^ Mahmoud et al. found that prostate cancer cells store and use cholesterol as energy.^13^ Cholesterol ester accumulation was observed in both pancreatic cancer samples and pancreatic cancer cell lines, indicating that there may be a correlation between cholesterol esterification and cancer cell metastasis.^11^ In addition, a study published in Cell Metabolism showed that high cholesterol levels upregulated the expression of T‐cell immune checkpoints, resulting in T‐cell function suppression and weakened antitumour immunity; the team also revealed the mechanism by which high cholesterol levels inhibit the immune function of T cells and found potential targets to rescue T cells and restore the killing effect.^14^ They further found that statins reduce cholesterol in the tumour microenvironment and alleviate the inhibitory effect of tumours on T cells, thereby promoting immunity. A study in Cancer Cell showed that glioma cells transport a large amount of cholesterol into tumour cells to maintain cell survival and that this mechanism can be specifically and effectively attenuated through small molecules to promote tumour cell death.^15^ In another study, cholesterol ester accumulation was found in pancreatic cancer tumour samples and cell lines, indicating that there may be a certain association between cholesterol esterification and tumour cells.

The results of the above studies indicate that lipids can modulate the antitumour effects of immune cells, potentially promoting the malignant progression of tumours. However, studies of lipids in pancreatic cancer are very rare, and in particular, it remains to be determined whether lipids can affect CD8+ T lymphocytes in tumour tissues. It is not known whether lipids can influence patient prognosis or immunotherapy efficacy by affecting the spatial infiltration of CD8+ T lymphocytes in tumour tissue. The aim of this study was to investigate the effect of plasma lipids on the prognosis of patients with pancreatic cancer and on the infiltration of CD8+ T lymphocytes in tumour tissues.

## 2 MATERIALS AND METHODS

### 2.1 Patients and tissue samples

We retrospectively analysed the data of 90 pancreatic cancer patients who underwent surgery at the Second Affiliated Hospital of Jiaxing University between 2012 and 2021. All patients with pancreatic cancer were treated with the first‐line chemotherapy regimen recommended by the guidelines. The postoperative pathological diagnosis was pancreatic ductal adenocarcinoma (PDAC). All patients had complete clinicopathological information and prognostic follow‐up data, including sex, age, grade, tumour size, tumour site, local invasion, perineural invasion, vascular invasion, lymph node metastasis, T stage, N stage, M stage, AJCC stage, smoking history, drinking history, diabetes history, survival status and survival time. In addition, plasma total cholesterol (TC) and triglyceride (TG) levels were measured before surgery. Cancer and paired paracancerous tissues were collected from 90 patients. A tissue microarray (TMA) was generated using the procedure described by Jawhar et al.^16^ The TMA had a total of 180 sites, that is, 90 tumour tissue sites and 90 paired paracancerous normal tissue sites. The TMA was used for subsequent immunohistochemistry (IHC) staining.

### 2.2 IHC and interpretation

TMA paraffin blocks were cut into 4‐μm sections, and the sections were deparaffinised in xylene and an ethanol gradient. Antigen retrieval was performed using citric acid, followed by staining with an anti‐CD8 alpha antibody (Maixin, MAB‐0021, 1:4000). Preliminary observation was performed under a microscope, and undesirable spots in the image during the preparation and staining process were eliminated. TMA readings were analysed by two independent pathologists (blinded to patient clinical data). The digitally scanned image files of the IHC chip were analysed using an Aperio ImageScope digital pathology system. IHC of CD8 was performed using the immunoreactive score (IRS) method. The IRS is the product of the staining intensity and percent of positive nuclei scores. In brief, the staining intensity score ranged from 0 to 2: 0 = no positivity, 1 = weak and 2 = moderate. The percent of positive nuclei score ranged from 0 to 4: 0 = 0% cells stained, 1 = 1%–10% cells stained, 2 = 11%–50% cells stained, 3 = 51%–80% cells stained (strong reaction) and 4 = 81%–100% cells stained. The IRS was calculated according to the following formula: IRS = staining intensity score×percentage of positive nuclei score. Hence, the IRS ranged from 0 to 12.

### 2.3 Statistical analysis

In this study, comparisons of measurement data were performed using *t*‐tests, and comparisons of qualitative data were performed using χ^2^ tests, the Fisher exact probability method or an R × C contingency table. Survival was analysed using the Kaplan–Meier method to generate survival curves, and the log‐rank method was used to compare differences in survival rates between groups. Multivariate analysis was performed using a Cox multivariate regression analysis model. The correlation between the blood lipid level and the level of CD8+ T‐cell infiltration was analysed using Spearman correlation analysis. In this study, *p* < 0.05 was considered statistically significant. The following graphing software packages were used in this study: SPSS 22.0, GraphPad Prism 6.02, Adobe Photoshop CS5 and Adobe Illustrator CS6.

## 3 RESULTS

### 3.1 Patient characteristics

A total of 90 PDAC patients were included in this study. The clinicopathological data of 90 patients are provided in Table 1.

**TABLE 1:** Patient demographics and clinicopathologic factors.

Factor	Value	No. of patients (%)
*n*		90
Sex (%)	Male	50 (55.6)
	Female	40 (44.4)
Age (%)	<65	45 (50.0)
	≥65	45 (50.0)
Grade (%)	1	40 (44.4)
	2	42 (46.7)
	3	8 (8.9)
Tumour size (%)	≤3 cm	47 (52.2)
	>3 cm	43 (47.8)
Tumour site (%)	Head	66 (73.3)
	Others	24 (26.7)
Local invasion (%)	No	40 (44.4)
	Yes	50 (55.6)
Perineural invasion (%)	No	11 (12.2)
	Yes	79 (87.8)
Vascular invasion (%)	No	61 (67.8)
	Yes	29 (32.2)
Lymph node metastasis (%)	No	51 (56.7)
	Yes	39 (43.3)
T (%)	1	16 (17.8)
	2	45 (50.0)
	3	27 (30.0)
	4	2 (2.2)
N (%)	0	51 (56.7)
	1	32 (35.6)
	2	7 (7.8)
M (%)	0	85 (94.4)
	1	5 (5.6)
AJCC stage (%)^a^	I	35 (38.9)
	II	42 (46.7)
	III	8 (8.9)
	IV	5 (5.6)
Smoking (%)	No	66 (73.3)
	Yes	24 (26.7)
Drinking (%)	No	73 (81.1)
	Yes	17 (18.9)
Diabetes (%)	No	78 (86.7)
	Yes	12 (13.3)

a TNM stage of patients with pancreatic adenocarcinoma assessed per the American Joint Commission on Cancer guidelines (8th edition).

### 3.2 Association between TC level and clinicopathological factors

The TC levels of all 90 patients were sorted from low to high. The 45 patients with low TC levels were distributed into the low TC group (3.37 ± 0.90 mmol/L), and the 45 patients with high TC levels were distributed into the high TC group (5.34 ± 1.20 mmol/L). Plasma TC levels were significantly correlated with tumour grade (*p* = 0.039) and lymph node metastasis (*p* = 0.033). Patients with high TC levels tended to have high tumour grades and were more likely to have lymph node metastasis (Table 2).

**TABLE 2:** Relationships between TC level and clinicopathologic factors.

Factor	Value	TC level		*P* values
		Low (%)	High (%)	
Sex (%)	Male	29 (64.4)	21 (46.7)	0.138
	Female	16 (35.6)	24 (53.3)	
Age (%)	<65	25 (55.6)	20 (44.4)	0.399
	≥65	20 (44.4)	25 (55.6)	
Grade (%)	1	14 (31.1)	26 (57.8)	0.039
	2	26 (57.8)	16 (35.6)	
	3	5 (11.1)	3 (6.7)	
Tumour size (%)	≤3 cm	25 (55.6)	22 (48.9)	0.673
	>3 cm	20 (44.4)	23 (51.1)	
Tumour site (%)	Head	33 (73.3)	33 (73.3)	1
	Others	12 (26.7)	12 (26.7)	
Local invasion (%)	No	19 (42.2)	21 (46.7)	0.832
	Yes	26 (57.8)	24 (53.3)	
Perineural invasion (%)	No	5 (11.1)	6 (13.3)	1
	Yes	40 (88.9)	39 (86.7)	
Vascular invasion (%)	No	34 (75.6)	27 (60.0)	0.176
	Yes	11 (24.4)	18 (40.0)	
Lymph node metastasis (%)	No	31 (68.9)	20 (44.4)	0.033
	Yes	14 (31.1)	25 (55.6)	
T (%)	1	7 (15.6)	9 (20.0)	0.519
	2	26 (57.8)	19 (42.2)	
	3	11 (24.4)	16 (35.6)	
	4	1 (2.2)	1 (2.2)	
N (%)	0	31 (68.9)	20 (44.4)	0.06
	1	11 (24.4)	21 (46.7)	
	2	3 (6.7)	4 (8.9)	
M (%)	0	44 (97.8)	41 (91.1)	0.357
	1	1 (2.2)	4 (8.9)	
AJCC stage (%)	I	22 (48.9)	13 (28.9)	0.174
	II	18 (40.0)	24 (53.3)	
	III	4 (8.9)	4 (8.9)	
	IV	1 (2.2)	4 (8.9)	
Smoking (%)	No	33 (73.3)	33 (73.3)	1
	Yes	12 (26.7)	12 (26.7)	
Drinking (%)	No	35 (77.8)	38 (84.4)	0.59
	Yes	10 (22.2)	7 (15.6)	
Diabetes (%)	No	38 (84.4)	40 (88.9)	0.756
	Yes	7 (15.6)	5 (11.1)	

### 3.3 Association between TG level and clinicopathological factors

The TG levels of all 90 patients were sorted from low to high. The 45 patients with low TG levels were distributed into the low TG group (0.89 ± 0.18 mmol/L), and the 45 patients with high TG levels were distributed into the high TG group (1.94 ± 0.72 mmol/L). Plasma TG levels were significantly correlated with perineural invasion (*p* = 0.01), and patients with high TG levels tended to develop neurological invasion (Table 3).

**TABLE 3:** Relationships between TG level and clinicopathological factors.

Factor	Value	TC Level		*P* values
		Low (%)	High (%)	
Sex (%)	Male	27 (60.0)	23 (51.1)	0.525
	Female	18 (40.0)	22 (48.9)	
Age (%)	<65	24 (53.3)	21 (46.7)	0.673
	≥65	21 (46.7)	24 (53.3)	
Grade (%)	1	16 (35.6)	24 (53.3)	0.106
	2	26 (57.8)	16 (35.6)	
	3	3 (6.7)	5 (11.1)	
Tumour size (%)	≤3 cm	24 (53.3)	23 (51.1)	1
	>3 cm	21 (46.7)	22 (48.9)	
Tumour site (%)	Head	32 (71.1)	34 (75.6)	0.812
	Others	13 (28.9)	11 (24.4)	
Local invasion (%)	No	20 (44.4)	20 (44.4)	1
	Yes	25 (55.6)	25 (55.6)	
Perineural invasion (%)	No	10 (22.2)	1 (2.2)	0.01
	Yes	35 (77.8)	44 (97.8)	
Vascular invasion (%)	No	31 (68.9)	30 (66.7)	1
	Yes	14 (31.1)	15 (33.3)	
Lymph node metastasis (%)	No	28 (62.2)	23 (51.1)	0.395
	Yes	17 (37.8)	22 (48.9)	
T (%)	1	11 (24.4)	5 (11.1)	0.162
	2	20 (44.4)	25 (55.6)	
	3	12 (26.7)	15 (33.3)	
	4	2 (4.4)	0 (0.0)	
N (%)	0	28 (62.2)	23 (51.1)	0.567
	1	14 (31.1)	18 (40.0)	
	2	3 (6.7)	4 (8.9)	
M (%)	0	42 (93.3)	43 (95.6)	1
	1	3 (6.7)	2 (4.4)	
AJCC stage (%)	I	19 (42.2)	16 (35.6)	0.84
	II	19 (42.2)	23 (51.1)	
	III	4 (8.9)	4 (8.9)	
	IV	3 (6.7)	2 (4.4)	
Smoking (%)	No	32 (71.1)	34 (75.6)	0.812
	Yes	13 (28.9)	11 (24.4)	
Drinking (%)	No	35 (77.8)	38 (84.4)	0.59
	Yes	10 (22.2)	7 (15.6)	
Diabetes (%)	No	37 (82.2)	41 (91.1)	0.352
	Yes	8 (17.8)	4 (8.9)	

### 3.4 Pancreatic cancer patients with high TC/high TG levels had a poor prognosis

**FIGURE 1:**
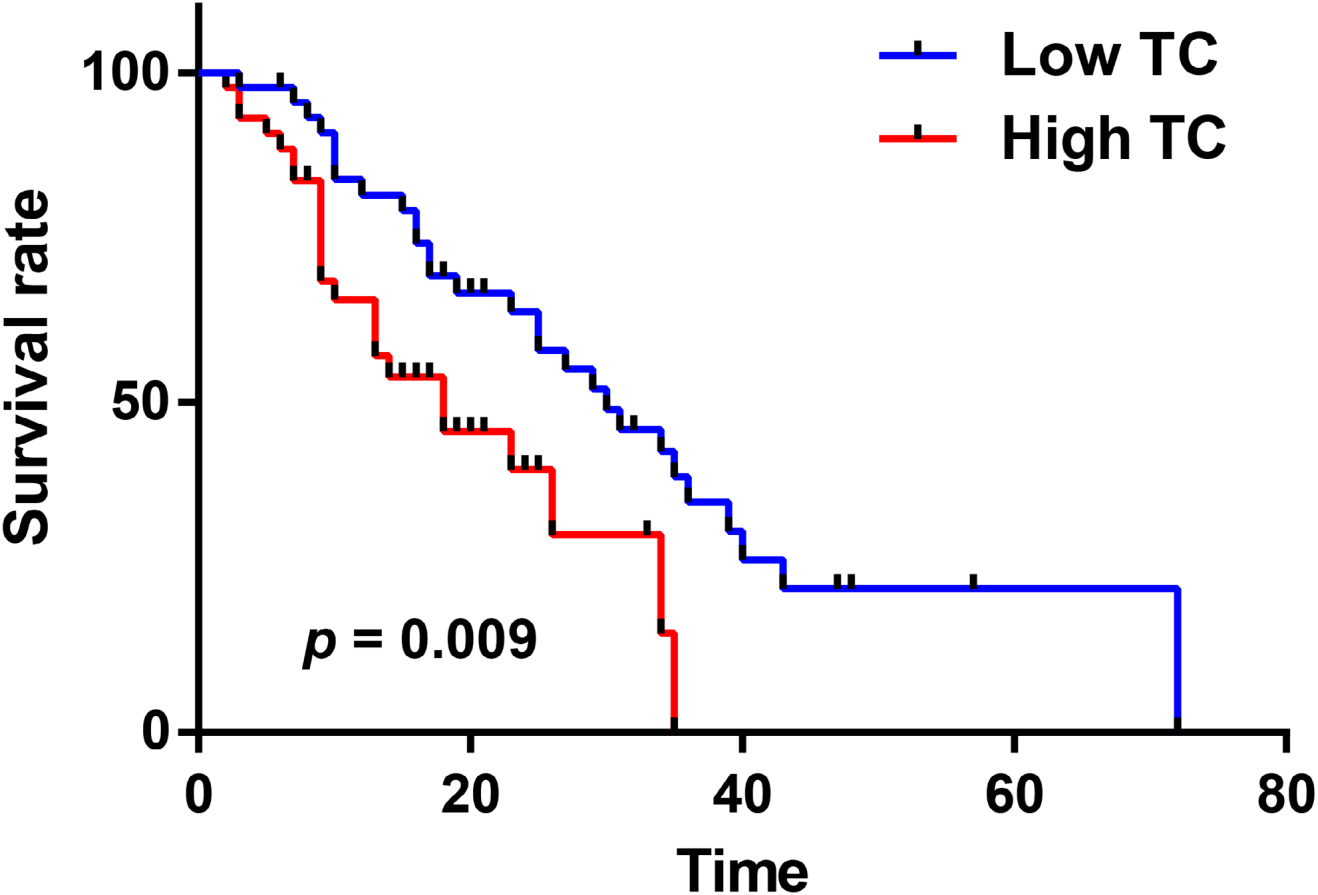
Kaplan–Meier survival curves for pancreatic cancer patients grouped based on TC level.

Overall survival (OS) was used as a prognostic indicator. The Kaplan–Meier survival analysis results indicated that the prognosis of patients in the high TC group was significantly worse than that of patients in the low TC group (*p* = 0.009, Figure 1). The median survival time of patients in the high TC group was 18 months, and the 95% confidence interval (CI) was 7.99–28.00 months. The median survival time of patients in the low TC group was 30 months, and the 95% CI was 20.95–39.05 months.

**FIGURE 2:**
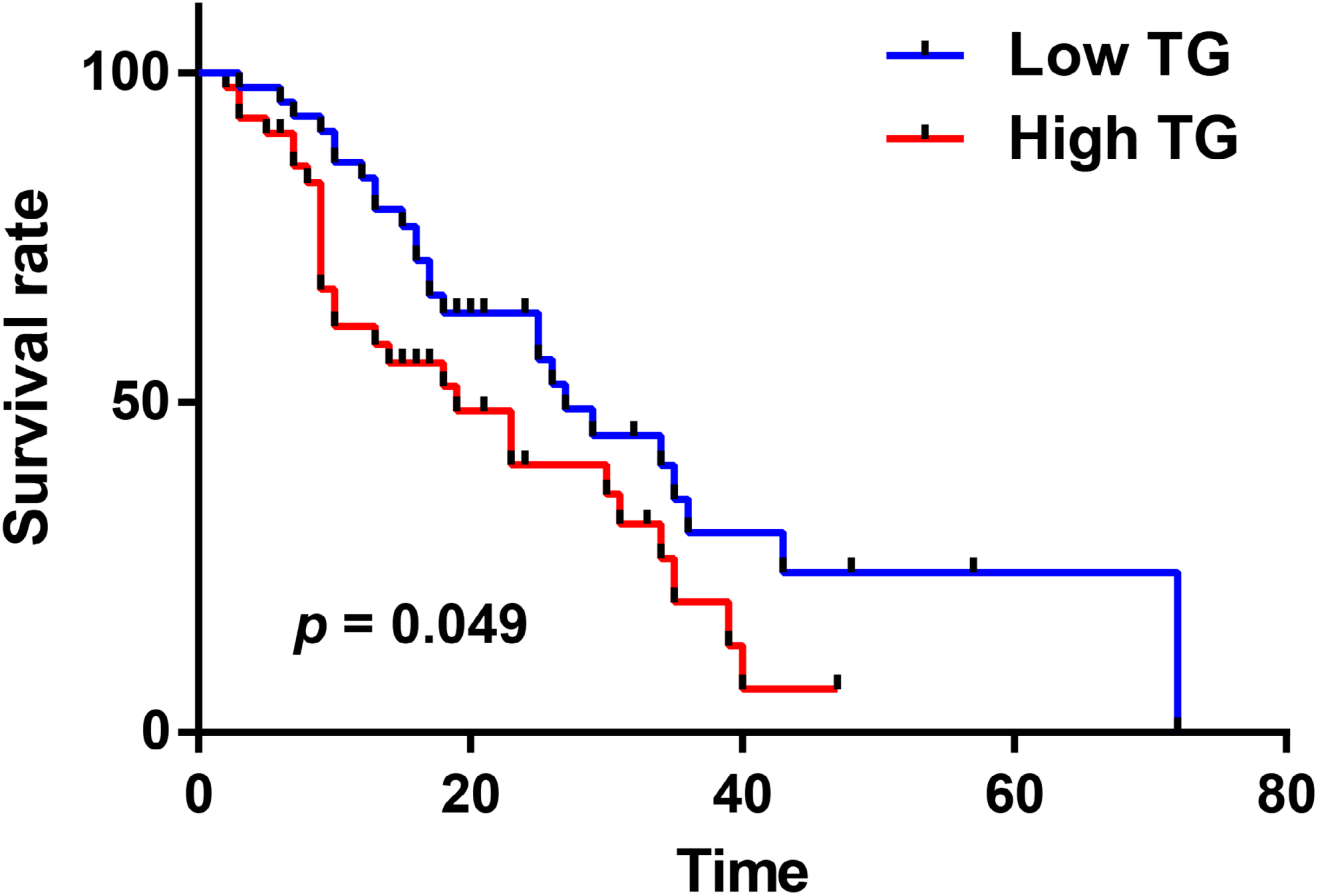
Kaplan–Meier survival curves for pancreatic cancer patients grouped based on TG level.

The Kaplan–Meier survival analysis results indicated that the prognosis of patients in the high TG group was significantly worse than that of patients in the low TG group (*p* = 0.049, Figure 2). The median survival time of patients in the high TG group was 19 months, and the 95% CI was 9.22–28.78 months. The median survival time of patients in the low TC group was 27 months, and the 95% CI was 21.15–32.85 months.

### 3.5 High TC levels were an unfavourable independent prognostic factor in patients with pancreatic cancer

Univariate survival analysis indicated that TC and TG were significantly correlated with the OS of pancreatic cancer patients and that high TC/TG levels were correlated with poor patient prognosis (*p* = 0.009, 0.049). The clinicopathological characteristics perineural invasion (*p* = 0.026), vascular invasion (*p* = 0.003), lymph node metastasis/N stage (*p* = 0.000), and T stage (*p* = 0.014) were significantly correlated with OS (*p* ≤ 0.05) and were associated with poor prognosis (Table 4).

**TABLE 4:** Univariate and multivariate analyses.

Factors	Value	Univariate analysis		Multivariate analysis		
		MST (Months)	*p* value	HR	95% CI	*p* value
Sex	Male/Female	25/25	0.973			
Age, years	<65/≥65	25/26	0.734			
Grade	I + II/III	16/18	0.339			
Tumour site	Head/Others	25/31	0.332			
Tumour size	≤3 cm/>3 cm	34/17	0.074	1.203	0.530–2.729	0.659
Local invasion	No/Yes	29/25	0.168			
Perineural invasion	No/Yes	72/23	0.026	2.032	0.661–6.243	0.216
Vascular invasion	No/Yes	30/16	0.003	1.650	0.842–3.232	0.145
Lymph node metastasis	No/Yes	34/14	0.000	2.485	1.332–4.638	0.004
T	T1 + T2/T3 + T4	30/16	0.014	1.503	0.640–3.526	0.350
N	N0/N1 + N2	34/14	0.000			
M	M0/M1	25/15	0.867			
AJCC stage	I + II/III + IV	26/16	0.290			
Smoking	No/Yes	26/18	0.573			
Drinking	No/Yes	26/23	0.922			
Diabetes	No/Yes	26/23	0.795			
TC	Low/High	27/19	0.049	1.991	1.045–3.790	0.036
TG	Low/High	30/18	0.009	1.292	0.718–2.323	0.393

Abbreviation: MST, median survival time.

The relevant clinicopathological factors in the above univariate analysis (*p* < 0.1 was the criterion for inclusion in the multivariate analysis), that is, tumour size, perineural invasion, vascular invasion, lymph node metastasis, T stage, TC and TG, were included in the Cox multivariate regression analysis. Cox multivariate regression indicated that lymph node metastasis (*p* = 0.004) and TC (*p* = 0.036) were independent prognostic factors for pancreatic cancer patients after surgery. Lymph node metastasis and TC were both unfavourable factors for the prognosis of pancreatic cancer patients, and the hazard ratios (HRs) were 2.485 (1.332–4.638) and 1.991 (1.045–3.790), respectively. TG may be affected by some indicators and was not ultimately identified as an independent prognostic factor for patients with pancreatic cancer, but the univariate analysis indicated that it was a significant factor.

### 3.6 Levels of CD8+ T cells in the TMA dataset

**FIGURE 3:**
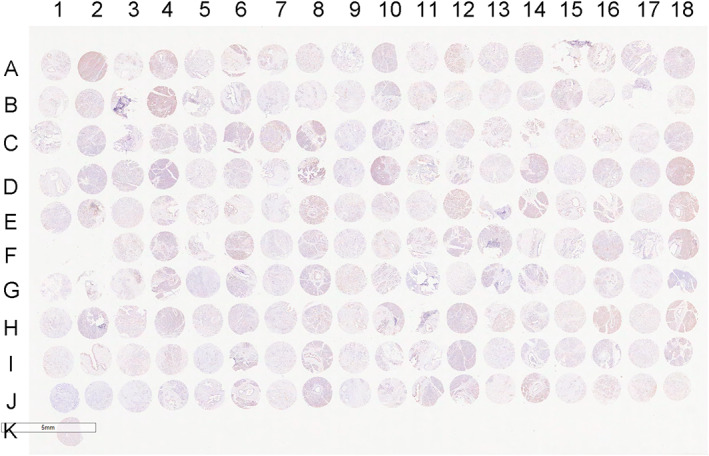
Digital image of the CD8 TMA. The TMA consisted of 90 tumour tissue samples and 90 paired paracancerous tissue samples from patients. The odd‐numbered columns were the intratumoural tumour tissues and the even‐numbered columns were their paired paracancerous tissue samples.

In this study, a pancreatic cancer TMA chip was constructed, including samples from a total of 90 patients. According to CD8 IHC staining and assessment of CD8+ T lymphocyte infiltration, the CD8 IRS in pancreatic cancer tissue was 4.27 ± 1.99; the lowest IRS was 1, and the highest IRS was 1.9 (Figure 3).

### 3.7 Analysis of the correlation of TC with CD8+ T‐cell infiltration

Spearman correlation analysis indicated that there was a significant negative correlation between CD8 IRS and TC (*r* = −0.725, *p* = 0.000, Table 5). The results showed that CD8+ T lymphocyte infiltration in pancreatic cancer tissue from patients with high TC was reduced. High TC levels may affect CD8+ T lymphocyte infiltration in tumour tissue and reduce the antitumour immune response.

**TABLE 5:** Analysis of the correlation of TC with CD8 T‐cell infiltration in pancreatic cancer tissue.

		CD8 IRS					
		1	2	3	4	6	9
TC	Low	0	0	4	6	32	3
	High	5	17	14	3	6	0
*r* *							−0.725
*p*							0.000

* Spearman's correlation.

### 3.8 Analysis of the correlation of TG with CD8+ T‐cell infiltration

Spearman correlation analysis indicated that there was a significant negative correlation between CD8 IRS and TG (*r* = −0.309, *p* = 0.003, Table 6). The results showed that there was less CD8+ T‐cell infiltration in pancreatic cancer tissue from patients with high TG levels. High TG levels may affect CD8+ T‐cell infiltration in tumour tissue, but this effect may not be as significant as the effect of TC.

**TABLE 6:** Analysis of the correlation of TG with CD8 T‐cell infiltration in pancreatic cancer tissue.

		CD8 IRS					
		1	2	3	4	6	9
TG	Low	1	6	6	5	25	2
	High	4	11	12	4	13	1
*r* *							−0.309
*p*							0.003

* Spearman's correlation.

## 4 DISCUSSION

Lifestyle‐related cancer risk factors include obesity, hyperlipidaemia, hypertension and hyperglycaemia, which affect the development and prognosis of cancer. Previous studies have concluded that hyperglycaemia and hyperlipidaemia are risk factors for pancreatic cancer.^17^ Moreover, these risk factors are controllable, and controlling these risk factors can potentially prevent the occurrence of pancreatic cancer. One meta‐analysis suggested that high cholesterol intake may increase the risk of pancreatic cancer, but the mechanism was not elucidated.^18^ Another study did not find a significant correlation. Because of the correlation between hyperlipidaemia and pancreatic cancer, some researchers have proposed that statins can reduce the risk of pancreatic cancer, but this remains controversial.^19^ Several studies, including meta‐analyses, have shown that the use of statins does not significantly reduce the risk of pancreatic cancer.^20^, ^21^, ^22^ By contrast, patients with pancreatic cancer who routinely take statins before surgery have a shorter postoperative survival period.^23^ A randomised case–control study by Carey et al. showed that the regular use of statins significantly reduced the risk of pancreatic cancer in men who smoked.^24^ Regarding the mechanism by which statins inhibit pancreatic cancer, some studies suggest that statins can induce cell cycle G1 arrest and inhibit DNA synthesis, while some suggest that statins promote tumour cell apoptosis.^25^ This antitumour effect of statins does not seem to be directly related to reduced blood lipid levels. In our study, we found that high cholesterol levels were significantly associated with high tumour grade and lymph node metastasis and that high TG levels were associated with increased nerve invasion. In the univariate survival analysis, TC and TG levels were both unfavourable prognostic factors for pancreatic cancer patients after surgery. Cox multivariate analysis revealed that TC was an independent prognostic factor for pancreatic cancer patients. The risk of death among patients with high TC levels was 1.991 times higher than that among patients with low TC levels. Although TG was not an independent prognostic factor in the Cox multivariate analysis, the median survival time of patients with low TG levels was 12 months longer than that of patients with high TG levels.

Previous studies have suggested that cancer is caused by the malignant proliferation of tumour cells. However, this idea does not consider the tumour microenvironment in which tumour cells are located. The tumour microenvironment is hypoxic, acidic and nutrient deficient, and these characteristics may lead to metabolic remodelling and thus promote tumour progression.^26^, ^27^ Conversely, metabolic remodelling may cause metabolic disorders in patients and further cause changes in the tumour microenvironment,^27^, ^28^ forming a vicious cycle. Abnormal lipid metabolism is one of the areas of tumour metabolism that has attracted widespread attention in recent years. On the basis of their structural/biological characteristics, lipids can be divided into cholesterol, TGs, phospholipids, fatty acids (FAs) and sphingolipids. In this study, we investigated the common serum factors TC and TGs to explore their effects on the tumour immune microenvironment.

Why does a high‐lipid environment affect tumour progression and prognosis, and what is the underlying mechanism? High‐lipid levels promote tumour progression by increasing the energy supply of tumour cells but are there more complex tumour‐promoting mechanisms? A study by Zhang et al. noted that there is a complex interaction between metabolic reprogramming of immune cells and liver cancer cells, but the molecular mechanism needs to be further explored.^29^ A study by Thysell et al.^30^ showed that the cholesterol level in the tumour cell lipid membrane in prostate cancer bone metastases was associated with cell migration and that high cholesterol levels were associated with cancer metastasis.^31^ From a pathological point of view, pancreatic fat infiltration is a risk factor for pancreatic intraepithelial neoplasias (PanINs) and PDAC.^32^ Chronic pancreatitis is an important risk factor for pancreatic cancer, and a long‐term increase in serum TC levels causes an increase in proinflammatory cytokine levels and aggravates pancreatic cancer resulting from chronic pancreatitis.^33^ In this study, high cholesterol levels were found to be associated with high tumour grade and an increased likelihood of lymph node metastasis, which supports the above view.

In addition, we believe that a high‐lipid microenvironment may affect the infiltration and distribution of immune cells and their functional status. The replacement of glucose metabolism by lipid metabolism in the tumour microenvironment may result in impaired effector T‐cell function.^34^ In addition, it may also increase the infiltration of regulatory T (Treg) cells, which inhibit antitumour immunity.^29^ However, lipids are the basic components of cells, and some studies have suggested that a lack of lipids can inhibit CD8+ T‐cell proliferation. This conclusion is clearly contradictory to the above view.^35^

Pancreatic cancer has a poor response to immunotherapy, and pancreatic tumours have immune infiltration characteristics of the immune desert and immune exclusion types.^11^ This indicates that a variety of factors that inhibit CD8+ T cells are present in the microenvironment. We speculate that lipid metabolism affects the infiltration of antitumour immune cells in pancreatic cancer tissues and antitumour and tumour cell‐killing functions. Therefore, in this study, we analysed the correlation between preoperative serum TC and TG and CD8+ T‐cell infiltration in tumour tissues in 90 patients who underwent pancreatic surgery; we found that TC and TG levels were negatively correlated with CD8+ T‐cell infiltration in tumour tissues and that the prognosis of patients with high TC and high TG levels was worse than that of those with low levels. We speculate that the abnormally high‐lipid level changes the immune microenvironment and reduces the infiltration of immune cells into the microenvironment, which may lead to poor immunotherapy efficacy in pancreatic cancer. It has been reported that aerobic glycolysis is the main pathway by which CD8+ T cells provide energy for cell growth and cytokine production.^36^ Therefore, it is reasonable to speculate that tumour‐infiltrating CD8+ T cells become dysfunctional in the high‐lipid, low‐glucose, hypoxic microenvironment, a process that may be related to CD8+ T lymphocyte‐mediated lipid uptake via CD36, leading to lymphocyte dysfunction.^37^ Unfortunately, in this study, we did not further investigate the function of CD8+ T cells in the high‐lipid microenvironment. In addition, the high‐lipid microenvironment may also affect the functions of other immune cells. For example, when natural killer (NK) cells become dysfunctional, the immune surveillance of tumours is impaired, resulting in immune escape and accelerated tumour progression.^38^

In addition, we found that patients with high TC had significantly more likely to have lymph node metastasis (*p* = 0.033). Lymph node metastasis may be related to the decrease in CD8+ T‐cell infiltration caused by high TC. These results suggest that there may be a close relationship between lymph node positivity and CD8+ T cells. This idea is supported by a study by Silvio Daster et al., who showed that a high frequency of CD8+ lymphocyte infiltration is significantly predictive of lymph node negativity in early rectal cancers.^39^ Taylor et al. reported that in a cohort of 875 melanoma patients, the presence of tumour‐infiltrating lymphocytes (TILs) was an independent predictor of negative sentinel lymph node metastasis.^40^ In cervical cancer, CD8+ T‐cell infiltration in tumour tissue is associated with a lower likelihood of lymph node metastasis and thus better prognosis.^41^ CD8+ T‐cell infiltration is one of the factors predicting lymph node positivity in prostate cancer.^42^ In our study, we hypothesised that high TC inhibited the effect of CD8+ T cells in reducing the likelihood of lymph node metastasis. Therefore, depletion of TC may enhance the functions of CD8+ T cells and reduce lymph node metastasis to prevent primary tumour immune escape. In summary, early TC reduction enhances immune surveillance.

## 5 CONCLUSIONS

TC and TG levels are significantly correlated with the prognosis of pancreatic cancer patients and may affect tumour progression to advanced grades, lymph node metastasis and nerve invasion. More importantly, TC and TGs may reduce the infiltration of CD8+ T lymphocytes into pancreatic cancer tissues, affecting antitumour immune function and the efficacy of immunotherapy. Therefore, further investigation of the mechanism by which high‐lipid levels affect the tumour immune microenvironment is warranted to improve the efficacy of pancreatic cancer immunotherapy.

## AUTHOR CONTRIBUTIONS

**Bin Wu:** Project administration (lead). **Weiai Shen:** Data curation (supporting). **Xiaoguang Wang:** Writing – original draft (lead). **Jing Wang:** Formal analysis (lead). **Zhengxiang Zhong:** Writing – review and editing (lead). **Zhongcheng Zhou:** Writing – review and editing (lead). **Xujian Chen:** Methodology (lead).

## FUNDING INFORMATION

The present study was supported by the Medical and Health Science and Technology Project from the Health Committee of Provincial Zhejiang (grant no. 2020KY951), the Medical and Health Science and Technology Project from the Health Committee of Provincial Zhejiang (grant no. 2023RC284) and project of the Bureau of Science and Technology of Jiaxing City (2020AY30017).

## CONFLICT OF INTEREST STATEMENT

The authors declare that the research was conducted in the absence of any commercial or financial relationships that could be construed as a potential conflict of interest.

## ETHICS STATEMENT

All patients or their guardians were informed of and agreed to the usage of the tissue/blood samples in our research.

## RESEARCH INVOLVING HUMAN PARTICIPANTS AND/OR ANIMALS

This article does not contain any studies with animals.

This research was approved by the Ethical Committee of the Second Affiliated Hospital of Jiaxing University, Jiaxing, China (Ethics Approval No. 009, 2023, the Second Hospital of Jiaxing).

## Data Availability

The data analysed in this article cannot be shared publicly to maintain the privacy of the individuals who participated in the study. The data will be shared upon reasonable request to the corresponding author.
